# Baseline Fatty Acids and Time‐Averaged Executive Function in Ugandan Children With Perinatal HIV

**DOI:** 10.1111/nyas.70374

**Published:** 2026-08-21

**Authors:** Vanessa N. Cardino, Bruno Giordani, Alla Sikorskii, Sarah K. Zalwango, Jenifer I. Fenton, Amara E. Ezeamama

**Affiliations:** ^1^ Department of Food Science and Human Nutrition Michigan State University East Lansing Michigan USA; ^2^ Departments of Psychiatry, Neurology, Psychology, and School of Nursing University of Michigan Ann Arbor Michigan USA; ^3^ Department of Psychiatry Michigan State University East Lansing Michigan USA; ^4^ Kampala Capital City Authority Kampala Uganda

**Keywords:** children, epidemiology, executive function, fatty acid, HIV/AIDS research, public health, serum biomarkers

## Abstract

Fatty acids (FAs) play critical neurodevelopmental roles, yet associations with executive function among children affected by perinatal HIV remain poorly characterized. This prospective cohort study examined associations between serum FAs and caregiver‐reported executive function and evaluated effect modification by perinatal HIV status among 6–10‐year‐old Ugandan children who had perinatal HIV infection (CPHIV; *n* = 84), were HIV exposed but uninfected (CHEU; *n* = 78), or were HIV unexposed and uninfected (CHUU; *n* = 81). Serum FAs were measured at enrollment, and executive dysfunction was assessed at enrollment, 6, and 12 months (Executive Functioning Index (EFI) and Global Executive Composite (GEC)). Multivariable linear mixed models estimated overall and HIV‐specific mean differences (MDs) and 95% confidence intervals (CIs) in executive dysfunction z‐scores by FA tertiles. Moderate versus low arachidonic acid (MD [95% CI] EFI: −0.40 [−0.71, −0.09]; GEC: −0.30 [−0.58, −0.03]) and highly unsaturated FAs (HUFAs; EFI: −0.41 [−0.72, −0.11]) associated with lower executive dysfunction. Among CPHIV, high Mead acid (20:3 (n‐9)) associated with higher executive dysfunction (EFI: 0.56 [0.11, 1.01]). Among CHEU, lower executive function was associated with high docosatetraenoic acid (EFI: −0.93 [−1.71, −0.15]) and docosahexaenoic acid (GEC: −0.78 [−1.26, −0.31]). These results showed that higher HUFAs associated with better executive function, particularly among CHEU, supporting further nutritional intervention research for children affected by perinatal HIV.

## Graphical abstract

This study examined serum fatty acids and executive function among Ugandan children with perinatal HIV infection (CPHIV), HIV‐exposed uninfected (CHEU), and HIV‐unexposed uninfected (CHUU). Higher total highly unsaturated fatty acid (HUFA) levels, notably arachidonic acid, were associated with lower executive dysfunction. Exploratory analyses showed higher select HUFA levels, notably ω‐6 docosatetraenoic acid and ω‐3 docosahexaenoic acid, were associated with less dysfunction among CHEU. These findings support beneficial HUFA–executive function associations.

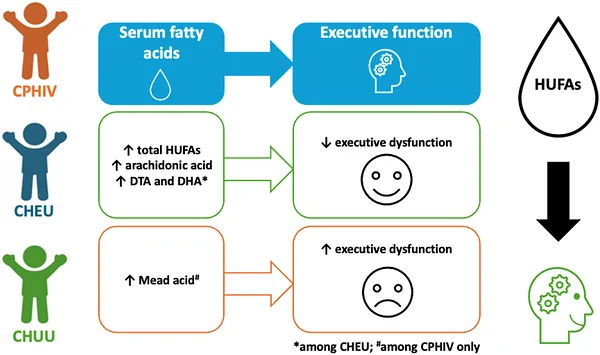

## 1 Introduction

Fatty acids (FAs) are essential for early‐life cognitive development and long‐term cognitive health. Saturated FAs (SFAs) are generally negatively associated with cognitive functioning, whereas monounsaturated FAs (MUFAs) and ω‐6 and ω‐3 polyunsaturated FAs (PUFAs) are positively associated with cognition [1, 2, 3, 4, 5, 6, 7, 8, 9, 10]. The essential FAs ω‐6 linoleic acid (LA) and ω‐3 α‐linolenic acid (ALA) and their highly unsaturated FA (HUFA) metabolites, notably ω‐3 eicosapentaenoic acid (EPA), ω‐3 docosahexaenoic acid (DHA), and ω‐6 arachidonic acid, support brain growth, myelination, synaptic function, and neuroinflammation regulation [11, 12, 13, 14, 15, 16, 17, 18, 19, 20, 21, 22].

To characterize FA status and inflammatory risk, studies commonly use composite markers. A high ω‐6:ω‐3 ratio may indicate high neuroinflammation, which is associated with poorer cognition [23]. The Omega‐3 Index and the HUFA ratio measure relative ω‐3 PUFA abundance [24, 25]. Essential FA deficiency (EFAD) is measured using Mead acid levels and the triene:tetraene (T:T) ratio, which increase as arachidonic acid decreases with low essential FA intake [26]. The measurement of FA levels in childhood is critical, as this period is key for cognitive development, particularly executive function.

Executive function comprises advanced skills supporting behavior regulation and problem solving, including inhibitory control, working memory, and cognitive flexibility [27, 28]. These skills can be assessed through caregiver‐ or teacher‐reported questionnaires that capture real‐world functioning [29, 30, 31]. Middle childhood (ages 6–10 years) is a sensitive period for executive function due to prefrontal cortex maturation and increasing integration of neural networks [32, 33, 34]. Although there is evidence of associations between FAs and executive function, they are limited to child populations under 6 years old, cross‐sectional study designs, and/or limited FA assessment [7, 8, 9, 17, 18]. These studies have also not examined these associations in populations affected by perinatal HIV.

Perinatal HIV exposure or infection may further impact executive function development [35]. Mechanistic evidence suggests cognitive vulnerability through immune dysregulation development, mitochondrial dysfunction, and impaired brain maturation, with epidemiologic support from both high‐ and low‐income countries [36, 37, 38, 39, 40]. Perinatal HIV exposure and infection may also disrupt FA metabolism through altered desaturase activity and intestinal epithelial damage, potentially impairing fat absorption [41, 42, 43, 44, 45]. Existing studies suggest children with perinatal HIV infection (CPHIV) have lower ω‐6 PUFA levels and higher EFAD markers than children perinatally unexposed to and uninfected with HIV (CHUU), though data on children perinatally exposed to but uninfected with HIV (CHEU) are limited [46, 47]. Children in middle childhood from Kampala, Uganda represent a key population to examine these relationships. Uganda has a high prevalence of CHEU (∼1 million) and CPHIV (∼72,000), alongside dietary patterns characterized by low PUFA intake due to reliance on staple grains and increasing consumption of ultra‐processed foods [35, 48].

The objectives of this study were to (1) determine time‐averaged associations between baseline serum FA levels and 12‐month proxy‐reported executive dysfunction among Ugandan children aged 6–10 years, and (2) assess whether these associations differ by perinatal HIV status. It was hypothesized that higher MUFAs and PUFAs would be associated with lower executive dysfunction, whereas higher SFAs, ω‐6:ω‐3 ratio, and EFAD markers would be associated with greater dysfunction, with strongest associations among CPHIV. Results from this study can be used to develop tailored nutrition interventions to improve the functional survival of children affected by perinatal HIV.

## 2 Materials and Methods

### 2.1 Study Design and Participants

This study is a secondary analysis of data from a 12‐month prospective cohort study of 6–10‐year‐old children from Kampala, Uganda (*n* = 306). As previously described [49, 50], three groups were recruited between March 15, 2017 and September 15, 2018 from Kawaala Health Center (KHC) based on confirmed perinatal HIV status at enrollment: (1) CPHIV, (2) CHEU, (3) and CHUU. Blood samples and neurocognitive data from participating children and their caregivers were collected at enrollment and repeated at 6‐month‐ and 12‐month‐follow‐up. To be included in this study, children needed to provide at least one viable serum sample (i.e., a minimum volume of 200 µL without visible hemolysis) at baseline. Additionally, children needed to complete at least one executive function assessment during at least one study visit.

### 2.2 Ethical Approval

The research ethics review boards of Michigan State University (IRB Protocol#: 16–828), Makerere University College of Health Sciences, School of Medicine (Protocol REC REF# 2017‐017) and the Uganda National Council for Science and Technology (Protocol #: SS 4378) approved the prospective cohort study protocols. Caregivers provided written informed consent, and all children provided assent, for their participation.

### 2.3 Exposure: Baseline Serum FAs

KHC study personnel collected fasted venous blood samples from child participants at enrollment. They deidentified the samples, separated serum from whole blood, and shipped serum samples on dry ice to Michigan State University for the methylation and quantification of FAs.

Serum FA methylation was done as described previously [51]. All reagents were acquired from Sigma Aldrich (St. Louis, MO, USA) unless otherwise specified. Briefly, serum samples were mixed with an internal standard (d‐35 stearic acid; Cayman Chemical, Ann Arbor, MI, USA) and a 1:4 v/v methanol chloride solution (with 0.1% butylated hydroxytoluene). They were then heated at 100°C for 1 h and 15 min to produce FA methyl esters (FAMEs). After neutralization with sodium bicarbonate, FAMEs were extracted using hexane, pooled, dried under nitrogen, and reconstituted in isooctane. A 200 µL aliquot was transferred to gas chromatography–mass spectroscopy (GC–MS) vials and stored at −20°C until analysis.

A Clarus 600/680 gas chromatograph/mass spectrometer (Perkin‐Elmer, Waltham, MA, USA) with a HP‐88 column (100 m length, 0.25 mm internal diameter, 0.2 µm film thickness; Agilent, Santa Clara, CA, USA) and helium carrier gas was used to quantify FAMEs. The GC method used was described previously [52]. Concisely, after a 14.5‐min solvent delay, the column was heated from 100°C to 240°C at 4°C/min and held for 20 min. The MS scanned *m*/*z* 70–400, and chromatograms were integrated using TargetLynx v4.0.1 (Waters Corporation, Milford, MA, USA). A total of 22 FAs were quantified and reported as a percentage of total FAs.

To evaluate prespecified FA patterns, primary exposures were defined a priori as (1) EFAD‐related markers (T:T ratio, Mead acid, and arachidonic acid) and (2) summary FA status measures reflecting FA metabolism and balance. These included HUFA ratio, total HUFA, Omega‐3 Index, total ω‐3 PUFA, total ω‐6 PUFA, ω‐6:ω‐3 ratio, total PUFA, total MUFA, and total SFA. Total PUFA was the sum of total ω‐6 PUFA, total ω‐3 PUFA, and Mead acid. The Omega‐3 Index was calculated using the following equation: (% serum EPA + % serum DHA + 0.0141)/0.7799 [53]. Total HUFA was calculated by summing dihomo‐γ‐linolenic acid (DGLA), arachidonic acid, docosatetraenoic acid (DTA), docosapentaenoic acid (DPA) ω‐6, EPA, DPA ω‐3, DHA, and Mead acid levels. The HUFA ratio was calculated as ω‐3 HUFAs divided by total HUFA multiplied by 100 [25]. The T:T ratio was calculated as the ratio of Mead acid to arachidonic acid. Individual FA levels were treated as secondary exposures of interest.

To compare relative differences in FA levels based on the knowledge of associations between relative FA levels and health outcomes [6, 54], each serum FA measure was divided into tertiles and transformed into a three‐level categorical variable. Categorization based on universal thresholds for FA sufficiency was not appropriate for this study population, as it resulted in uneven group distributions [54, 55]. To address this, tertiles were derived from the sample distribution to define population‐specific thresholds and ensure balanced classification. Tertile 1 was defined as the “low” level and was used as the reference category in main effect analyses. Tertile 2 was defined as the “moderate” level, and Tertile 3 was defined as the “high” level.

### 2.4 Outcome: Proxy‐Reported Executive Function

Due to the young age of study participants, executive function was measured via proxy‐reported questionnaires. Trained interviewers administered culturally adapted questionnaires to caregivers following a standard procedure at baseline, 6‐month, and 12‐month‐follow‐up as reported previously, and all executive function measures were transformed into age‐ and sex‐standardized z‐scores using CHUU as the reference population using the following formula: z‐score = (score − mean_CHUU of same age/sex at baseline_/(standard deviation_CHUU of same age/sex at baseline_) [50]. Two executive function questionnaires were used: the Behavior Assessment System for Children‐Third Edition (BASC‐3) and the Behavior Rating Inventory of Executive Functioning (BRIEF).

The BASC‐3 is a questionnaire administered to caregivers to assess their child's emotional and behavioral skills, and one of the domains measured is executive function impairment (i.e., executive dysfunction) [56]. Scores from 29 questions measuring four different executive function skills (problem solving, emotional control, attentional control, and behavioral control) were added to produce the Executive Functioning Index (EFI) score. A higher EFI z‐score indicated increased executive dysfunction.

The BRIEF proxy‐report questionnaire assesses executive dysfunction through questions that map to eight known executive function skills: working memory, task initiation, planning, monitoring, material organization, inhibition, flexibility, and emotional control [57]. The shortened 24‐question version was used to decrease caregiver burden and increase cultural acceptability [58]. It was adapted for the Ugandan context, including forward and backward translation to Luganda, as described previously [50]. Specifically, different reviewers administered the shortened BRIEF to 30 caregivers between 2 and 3 weeks apart to determine internal consistency (Cronbach's α = 0.89) and test–retest reliability (intraclass correlation [ICC] = 0.84–0.90), which were both excellent. The scores from each skill were summed to produce the Global Executive Composite (GEC) score, and a higher GEC z‐score was indicative of increased executive dysfunction.

### 2.5 Other Variables

Child age (in years) and biological sex (male vs. female) was provided by caregivers at enrollment. Caregivers self‐reported their sex and years of education at enrollment. Caregiver depressive symptoms were self‐reported using the 15 depression‐related questions from the Hopkins Symptom Checklist. The maximum score was 60, and a higher score indicated more symptoms [59]. Caregiver depression symptoms were transformed into a four‐level categorical variable using quartile distribution.

### 2.6 Statistical Analysis

Baseline demographic variables and proxy‐reported executive function z‐scores were summarized by perinatal HIV status using descriptive statistics. Differences in continuous variables were determined using one‐way analysis of variance (ANOVA) tests and were reported as means with standard deviations (*SD*s). Differences in categorical variables were determined using chi‐squared tests and were reported as frequencies with percentages.

Linear models and linear mixed‐effect models were used to estimate crude and adjusted time‐averaged mean differences (MDs) in executive function over 12 months according to baseline serum FA tertile, respectively. Preliminary linear mixed‐effect models included a time × FA interaction variable to assess nonlinear associations overtime. Effect modification by perinatal HIV status was evaluated in exploratory analyses using a perinatal HIV status × FA interaction term. Evidence of heterogeneity was defined descriptively as an interaction *p* value < 0.10, and corresponding HIV‐specific associations were estimated using pairwise comparisons analysis. These effect modification analyses were considered exploratory and were not adjusted for multiple comparisons. Multivariable models were controlled for confounders based on subject matter knowledge, notably the fixed effects of time, perinatal HIV status, caregiver sex, caregiver education years, and caregiver depression and the random effects of household and child ID. Additional sensitivity analyses, including modeling FA measures as continuous exposures and further adjusting main models for height‐for‐age z‐score (HAZ), were performed to evaluate the robustness of the main findings. All models were fit using complete‐case observations.

Crude and adjusted MDs and their robust 95% confidence intervals (CIs) are reported here. All hypothesis tests were considered statistically significant if α ≤ 0.05. As MD is similar to effect size, statistical and clinical significance could be interpreted. According to previous studies [50, 60], |MD| < 0.33 signals small/modest clinical importance, 0.33 ≤ |MD| ≤ 0.50 signals moderate clinical importance, and |MD| > 0.50 signals large clinical importance. R (Vienna, Austria), with the following packages, was used to conduct all analyses: *lme4*, *lmtest*, *lmerTest*, *car*, *emmeans*, *sandwich*, *clubSandwich*, and *multiwayvcov*.

## 3 Results

### 3.1 Baseline Characteristics and Global Executive Function by Perinatal HIV Status

Of the 306 children originally enrolled in the cohort study, 243 met the eligibility criteria for this analysis (CPHIV *n* = 84; CHEU *n* = 78; CHUU *n* = 81; Table 1). The study population exhibited an average age of 7.70 (*SD*: 1.46) years and was 47% female. Only caregiver depression differed significantly by perinatal HIV status; caregivers of CPHIV exhibited lower depression (mean [*SD*]: 9.05 [7.04]) than those of CHEU (13.88 [7.81]) and CHUU (13.33 [8.20]; *p* < 0.01).

**TABLE 1:** Baseline characteristics by perinatal HIV status among 6–10‐year‐old Ugandan children.

Characteristic	Total		CPHIV		CHEU		CHUU		*p* value
**Child characteristic**	***n***	**Mean (*SD*)**	***n***	**Mean (*SD*)**	***n***	**Mean (*SD*)**	***n***	**Mean (*SD*)**	
Age (years)	243	7.70 (1.46)	84	7.82 (1.55)	78	7.47 (1.34)	81	7.80 (1.48)	0.92
	***n***	**%**	***n***	**%**	***n***	**%**	***n***	**%**	
Sex female	115	47	40	48	41	53	34	42	0.41
**Caregiver characteristic**	***n***	**Mean (*SD*)**	***n***	**Mean (*SD*)**	***n***	**Mean (*SD*)**	***n***	**Mean (*SD*)**	
Education (years)	233	4.43 (3.52)	78	4.16 (3.42)	75	4.33 (3.72)	80	4.85 (3.45)	0.26
	***n***	**%**	***n***	**%**	***n***	**%**	***n***	**%**	
Sex female	216	92	72	92	71	93	73	91	0.88
	***n***	**Mean (*SD*)**	***n***	**Mean (*SD*)**	***n***	**Mean (*SD*)**	***n***	**Mean (*SD*)**	
Depressive symptoms	235	12.01 (7.96)	82	9.05 (7.04)	75	13.88 (7.81)	78	13.33 (8.20)	**< 0.01**

*Note*: The bold values indicate a significant *p*‐value (*p* < 0.05).

Abbreviations: CHEU, children perinatally exposed to but uninfected with HIV; CHUU, children perinatally unexposed to and uninfected with HIV; CPHIV, children perinatally infected with HIV; *SD*, standard deviation.

### 3.2 Executive Function Across Study Visits

Both mean EFI (baseline mean (*SD*): −0.15 [1.21]; 6‐month visit: −0.36 [1.17]; 12‐month visit −0.55 [1.07]) and GEC (baseline mean (*SD*): −0.10 [1.05]; 6‐month visit: −0.12 [1.02]; 12‐month visit −0.37 [0.91]) z‐scores decreased significantly over time, indicating lower executive dysfunction (all *p* < 0.01; Table 2). The number of children who completed EFI assessment decreased steadily across study visits (baseline: *n* = 236; 6‐month visit: *n* = 222; 12‐month visit: *n* = 217), whereas more children completed the GEC assessments at the 6‐month visit (*n* = 224) than at the baseline (*n* = 207) and 12‐month visit (*n* = 209).

**TABLE 2:** Mean executive function z‐scores across study visits.

	Baseline		6‐month visit		12‐month visit		*p* value
Executive function z‐score	*n*	Mean (*SD*)	*n*	Mean (*SD*)	*n*	Mean (*SD*)	
Executive Functioning Index z‐score	236	−0.15 (1.21)	222	−0.36 (1.17)	217	−0.55 (1.07)	**< 0.01**
Global Executive Composite z‐score	207	−0.10 (1.05)	224	−0.12 (1.02)	209	−0.37 (0.91)	**< 0.01**

*Note*: The bold values indicate a significant *p*‐value (*p* < 0.05).

Abbreviation: *SD*, standard deviation.

### 3.3 FA Tertiles by Perinatal HIV Status

The ranges of FA tertiles and the comparison of the frequency of participants in each tertile by perinatal HIV status are exhibited in Table S1. CPHIV were more likely to exhibit higher Mead acid levels, total HUFA levels, select ω‐6 PUFAs (i.e., γ‐linolenic acid, DGLA, DTA, and DPA ω‐6), and select SFA levels (i.e., stearic acid and arachidic acid) than CHEU and CHUU (all *p* < 0.05).

### 3.4 Differences in Global Executive Function z‐Scores by FA Tertile

Unadjusted for any potential confounders, those with moderate total HUFA levels exhibited significant decreases in both EFI and GEC z‐score compared to those with low levels, and a moderate and high versus low HUFA ratio was associated with a decrease in GEC z‐score (Table 3). In contrast, moderate versus low total PUFA levels were associated with an increase in EFI z‐score, and a high versus low ω‐6:ω‐3 ratio was associated with an increase in GEC z‐score. Moreover, crude analyses showed that moderate versus low total ω‐3 PUFAs, Omega‐3 Index, and DHA predicted decreases in EFI and GEC z‐scores. Moderate versus low total ω‐6 PUFAs were associated with an increased EFI z‐score, whereas moderate and high versus low arachidonic acid were associated with decreased EFI and GEC z‐scores.

**TABLE 3:** Time‐averaged mean differences in global executive function z‐scores relative to low serum fatty acid tertile among 6–10‐year‐old Ugandan children.

Fatty acid		BASC proxy‐report Executive Functioning Index z‐score (*n* = 232)		BRIEF proxy‐report Global Executive Composite z‐score (*n* = 232)	
	Tertile	Crude model	Adjusted model^a^	Crude model	Adjusted model^a^
		MD (95% CI)	MD (95% CI)	MD (95% CI)	MD (95% CI)
EFAD markers					
T:T ratio	Moderate	0.14 (−0.07, 0.35)	—	0.02 (−0.17, 0.20)	0.01 (−0.26, 0.27)
	High	−0.12 (−0.34, 0.10)	—	−0.08 (−0.28, 0.12)	−0.06 (−0.35, 0.23)
Mead	Moderate	0.09 (−0.12, 0.30)	—	−0.02 (−0.21, 0.16)	−0.02 (−0.29, 0.24)
	High	−0.04 (−0.27, 0.18)	—	−0.07 (−0.27, 0.12)	−0.05 (−0.36, 0.26)
Arachidonic	Moderate	−0.37 (−0.58, −0.16)	−**0.40 (**−**0.71,** −**0.09)**	−0.32 (−0.51, −0.13)	−**0.30 (**−**0.58,** −**0.03)**
	High	−0.24 (−0.46, −0.02)	−0.23 (−0.55, 0.10)	−0.31 (−0.50, −0.12)	−**0.31 (**−**0.61,** −**0.01)**
Sums/ratios					
HUFA ratio	Moderate	−0.20 (−0.42, 0.01)	−0.15 (−0.45, 0.15)	−0.25 (−0.45, −0.06)	−0.16 (−0.45, 0.13)
	High	−0.07 (−0.30, 0.16)	−0.03 (−0.38, 0.32)	−0.21 (−0.41, −0.02)	−0.09 (−0.38, 0.20)
Total HUFA	Moderate	−0.45 (−0.66, −0.23)	−**0.41 (**−**0.72,** −**0.11)**	−0.31 (−0.50, −0.12)	−0.26 (−0.55, 0.03)
	High	−0.19 (−0.42, 0.03)	−0.19 (−0.54, 0.15)	−0.24 (−0.43, −0.05)	−0.24 (−0.56, 0.08)
Omega‐3 Index	Moderate	−0.25 (−0.47, −0.04)	−0.23 (−0.53, 0.06)	−0.20 (−0.40, −0.01)	−0.13 (−0.41, 0.14)
	High	−0.11 (−0.33, 0.11)	−0.09 (−0.41, 0.23)	−0.24 (−0.43, −0.05)	−0.15 (−0.43, 0.13)
Total ω‐3 PUFA	Moderate	−0.30 (−0.52, −0.08)	−0.25 (−0.54, 0.05)	−0.31 (−0.50, −0.11)	−0.20 (−0.47, 0.07)
	High	−0.09 (−0.30, 0.13)	−0.08 (−0.39, 0.24)	−0.29 (−0.48, −0.10)	−0.21 (−0.50, 0.08)
Total ω‐6 PUFA	Moderate	0.33 (0.11, 0.55)	—	−0.06 (−0.26, 0.13)	−0.06 (−0.33, 0.22)
	High	0.15 (−0.08, 0.37)	—	0.07 (−0.12, 0.27)	0.07 (−0.21, 0.35)
ω‐6:ω‐3 ratio	Moderate	0.03 (−0.20, 0.26)	0.03 (−0.32, 0.38)	0.13 (−0.06, 0.33)	—
	High	0.18 (−0.04, 0.40)	0.15 (−0.18, 0.48)	0.33 (0.14, 0.52)	—
Total PUFA	Moderate	0.28 (0.06, 0.50)	—	0.09 (−0.10, 0.28)	0.06 (−0.22, 0.33)
	High	0.11 (−0.11, 0.34)	—	0.00 (−0.19, 0.19)	0.01 (−0.25, 0.28)
Total MUFA	Moderate	0.02 (−0.19, 0.23)	0.00 (−0.30, 0.29)	0.12 (−0.07, 0.31)	0.06 (−0.20, 0.32)
	High	−0.19 (−0.42, 0.03)	−0.13 (−0.47, 0.20)	0.02 (−0.18, 0.21)	0.02 (−0.27, 0.32)
Total SFA	Moderate	0.06 (−0.15, 0.28)	0.07 (−0.25, 0.38)	−0.01 (−0.21, 0.19)	−0.02 (−0.29, 0.25)
	High	−0.06 (−0.29, 0.17)	−0.03 (−0.35, 0.30)	0.03 (−0.16, 0.22)	0.05 (−0.22, 0.31)
ω‐3 PUFA					
ALA	Moderate	−0.13 (−0.35, 0.09)	−0.14 (−0.46, 0.17)	0.10 (−0.09, 0.28)	—
	High	0.01 (−0.21, 0.23)	0.01 (−0.32, 0.33)	−0.02 (−0.22, 0.18)	—
EPA	Moderate	0.00 (−0.22, 0.21)	0.00 (−0.34, 0.34)	−0.03 (−0.22, 0.16)	0.00 (−0.31, 0.30)
	High	0.07 (−0.15, 0.30)	0.10 (−0.25, 0.44)	−0.10 (−0.30, 0.09)	−0.04 (−0.34, 0.25)
DPA ω‐3	Moderate	0.02 (−0.20, 0.24)	0.10 (−0.20, 0.40)	0.03 (−0.17, 0.22)	0.08 (−0.18, 0.35)
	High	−0.05 (−0.28, 0.17)	0.04 (−0.30, 0.38)	−0.12 (−0.31, 0.07)	−0.06 (−0.34, 0.23)
DHA	Moderate	−0.24 (−0.45, −0.02)	−0.20 (−0.49, 0.10)	−0.31 (−0.51, −0.11)	—
	High	−0.19 (−0.41, 0.03)	−0.17 (−0.50, 0.15)	−0.34 (−0.53, −0.15)	—
ω‐6 PUFA					
LA	Moderate	0.08 (−0.15, 0.30)	—	0.00 (−0.20, 0.19)	−0.03 (−0.32, 0.26)
	High	0.06 (−0.17, 0.28)	—	0.14 (−0.06, 0.34)	0.13 (−0.18, 0.43)
GLA	Moderate	−0.05 (−0.28, 0.18)	−0.04 (−0.36, 0.28)	0.08 (−0.11, 0.27)	0.03 (−0.27, 0.33)
	High	−0.01 (−0.22, 0.20)	0.05 (−0.25, 0.35)	−0.06 (−0.26, 0.13)	−0.06 (−0.35, 0.23)
Eicosadienoic	Moderate	−0.03 (−0.24, 0.18)	—	−0.01 (−0.19, 0.18)	0.03 (−0.22, 0.28)
	High	−0.17 (−0.40, 0.06)	—	−0.15 (−0.35, 0.05)	−0.16 (−0.44, 0.13)
DGLA	Moderate	−0.18 (−0.39, 0.03)	−0.13 (−0.44, 0.17)	−0.10 (−0.28, 0.09)	−0.05 (−0.31, 0.20)
	High	−0.22 (−0.46, 0.01)	−0.12 (−0.52, 0.29)	−0.19 (−0.39, 0.01)	−0.17 (−0.51, 0.17)
DTA	Moderate	−0.08 (−0.30, 0.13)	—	0.07 (−0.12, 0.26)	—
	High	−0.25 (−0.47, −0.02)	—	−0.12 (−0.32, 0.07)	—
DPA ω‐6	Moderate	−0.04 (−0.27, 0.18)	−0.01 (−0.32, 0.31)	−0.06 (−0.25, 0.14)	−0.03 (−0.29, 0.22)
	High	−0.26 (−0.48, −0.04)	−0.17 (−0.51, 0.18)	−0.30 (−0.49, −0.11)	−0.25 (−0.55, 0.05)
MUFA					
Palmitoleic	Moderate	0.00 (−0.21, 0.21)	−0.02 (−0.33, 0.30)	0.08 (−0.12, 0.27)	0.05 (−0.21, 0.31)
	High	−0.16 (−0.39, 0.07)	−0.13 (−0.48, 0.21)	0.17 (−0.04, 0.38)	0.18 (−0.12, 0.48)
Palmitelaidic	Moderate	0.17 (−0.05, 0.38)	0.14 (−0.16, 0.45)	0.12 (−0.07, 0.31)	0.04 (−0.24, 0.31)
	High	−0.13 (−0.35, 0.10)	−0.05 (−0.40, 0.29)	−0.09 (−0.29, 0.11)	−0.08 (−0.37, 0.21)
Oleic	Moderate	−0.02 (−0.23, 0.20)	−0.06 (−0.37, 0.25)	0.07 (−0.12, 0.27)	0.04 (−0.24, 0.31)
	High	−0.15 (−0.37, 0.08)	−0.14 (−0.46, 0.18)	−0.01 (−0.21, 0.18)	−0.01 (−0.30, 0.28)
Eicosenoic	Moderate	0.03 (−0.18, 0.24)	0.10 (−0.2, 0.41)	0.07 (−0.12, 0.27)	0.11 (−0.18, 0.41)
	High	−0.04 (−0.26, 0.18)	−0.07 (−0.39, 0.26)	0.00 (−0.19, 0.19)	−0.04 (−0.33, 0.26)
Nervonic	Moderate	−0.18 (−0.39, 0.04)	−0.13 (−0.43, 0.17)	−0.05 (−0.24, 0.13)	0.00 (−0.28, 0.27)
	High	−0.01 (−0.23, 0.21)	0.02 (−0.29, 0.33)	0.11 (−0.08, 0.31)	0.15 (−0.12, 0.43)
SFA					
Myristic	Moderate	−0.05 (−0.27, 0.17)	0.02 (−0.30, 0.35)	−0.18 (−0.38, 0.02)	−0.20 (−0.47, 0.07)
	High	−0.29 (−0.52, −0.06)	−0.17 (−0.51, 0.17)	−0.17 (−0.37, 0.02)	−0.11 (−0.40, 0.17)
Palmitic	Moderate	−0.04 (−0.25, 0.17)	0.00 (−0.31, 0.32)	−0.07 (−0.27, 0.12)	−0.02 (−0.29, 0.25)
	High	0.04 (−0.19, 0.28)	0.04 (−0.30, 0.38)	0.10 (−0.10, 0.30)	0.10 (−0.17, 0.38)
Stearic	Moderate	−0.07 (−0.29, 0.15)	—	−0.07 (−0.26, 0.12)	−0.15 (−0.43, 0.13)
	High	−0.11 (−0.34, 0.11)	—	−0.05 (−0.25, 0.14)	−0.07 (−0.35, 0.22)
Arachidic	Moderate	−0.04 (−0.25, 0.18)	0.00 (−0.30, 0.30)	0.13 (−0.06, 0.32)	—
	High	−0.20 (−0.42, 0.02)	−0.18 (−0.49, 0.13)	0.02 (−0.18, 0.22)	—
Behenic	Moderate	−0.09 (−0.31, 0.14)	−0.03 (−0.36, 0.30)	0.18 (−0.01, 0.37)	0.17 (−0.09, 0.44)
	High	−0.22 (−0.43, 0.00)	−0.21 (−0.52, 0.11)	0.07 (−0.12, 0.26)	0.03 (−0.25, 0.30)
Lignoceric	Moderate	−0.01 (−0.23, 0.22)	−0.02 (−0.33, 0.29)	0.04 (−0.15, 0.24)	0.04 (−0.23, 0.31)
	High	−0.01 (−0.24, 0.21)	0.04 (−0.27, 0.36)	0.04 (−0.16, 0.24)	0.07 (−0.21, 0.35)

*Note*: The bold values indicate a significant mean difference (i.e., the confidence interval does not cross the null value).

Abbreviations: ALA, α‐linolenic acid; BASC, Behavior Assessment System for Children; BRIEF, Behavior Rating Inventory for Executive Function; CI, confidence interval; DGLA, dihomo‐γ‐linolenic acid; DHA, docosahexaenoic acid; DPA, docosapentaenoic acid; DTA, docosatetraenoic acid; EFAD, essential fatty acid deficiency; EPA, eicosapentaenoic acid; GLA, γ‐linolenic acid; HUFA, highly unsaturated fatty acid; LA, linoleic acid; MD, mean difference; MUFA, monounsaturated fatty acid; PUFA, polyunsaturated fatty acid; SFA, saturated fatty acid; T:T ratio, triene:tetraene ratio.

^a^ Missing values represent associations that had a significant perinatal HIV status × fatty acid (*p* < 0.10).

Following adjustment, moderate versus total HUFA (MD [95% CI]: −0.41 [−0.72, −0.11]) and arachidonic acid (−0.40 [−0.71, −0.09]) levels remained associated with an intermediate decrease in EFI z‐score. Both moderate (−0.30 [−0.58, −0.03]) and high (−0.31 [−0.61, −0.01]) arachidonic acid levels still predicted intermediate decreases in GEC z‐score. Heterogeneity testing determined that the associations between the following FAs and EFI z‐score differed by perinatal HIV status: T:T ratio, Mead acid, total ω‐6 PUFA, total PUFA, LA, eicosadienoic acid, DTA, and stearic acid. The associations between the ω‐6:ω‐3 ratio, ALA, DHA, DTA, and arachidic acid and GEC z‐score were found to significantly differ by perinatal HIV status following heterogeneity testing.

### 3.5 FA Tertile‐Associated Differences in Global Executive Function z‐Scores Modified by Perinatal HIV Status

**FIGURE 1:**
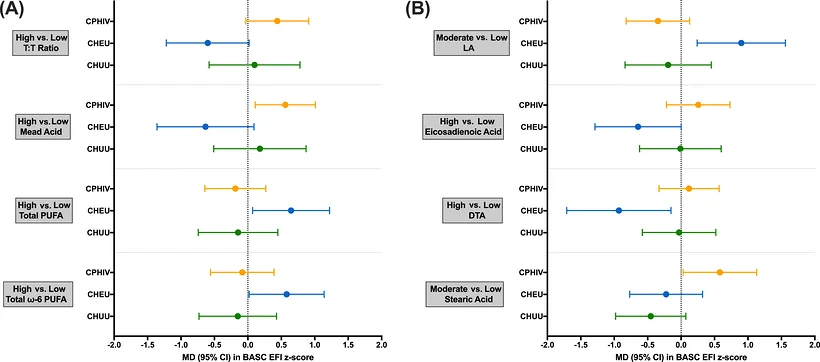
Time‐averaged HIV‐specific mean differences in proxy‐report Executive Functioning Index z‐score by fatty acid tertiles. (A) Comparison of serum EFAD marker or summary measure tertiles. (B) Comparison of individual serum ω‐6 PUFA or SFA measure tertiles. BASC, Behavior Assessment System for Children; CHEU, children perinatally exposed to but uninfected with HIV; CHUU, children perinatally unexposed to and uninfected with HIV; CI, confidence interval; CPHIV, children with perinatal HIV infection; DTA, docosatetraenoic acid; EFI, Executive Functioning Index; LA, linoleic acid; MD, mean difference; PUFA, polyunsaturated fatty acid; T:T ratio, triene:tetraene ratio.

The exploratory differences in EFI z‐score associated with FA tertile by perinatal HIV status are shown in Figure 1. Among CPHIV, a high versus low T:T ratio (MD [95% CI]: 0.44 [−0.03, 0.91]) and Mead acid levels (0.56 [0.11, 1.01]) were significantly associated or trended toward association with increases in EFI z‐score (Figure 1A). In contrast, CHEU exhibited trending negative associations, and CHUU demonstrated null associations, between EFAD markers and EFI z‐score. CHEU with high total PUFAs (0.65 [0.07, 1.22]) and total ω‐6 PUFAs (0.58 [0.02, 1.14]) showed strong increases in EFI z‐score compared to those with low levels, whereas CPHIV and CHUU exhibited trending negative associations.

Additionally, among CHEU, moderate versus low LA levels predicted large increases in EFI z‐score (MD [95% CI]: 0.90 [0.24, 1.56]), whereas they exhibited a trending association with decreased EFI z‐score among CPHIV and CHUU (Figure 1B). High versus low levels of eicosadienoic acid (−0.64 [−1.29, 0.00]) and DTA (−0.93 [−1.71, −0.15]) were associated or trended toward association with strong decreases in EFI z‐score among CHEU, whereas there were null or insignificant positive associations between these FAs and EFI z‐score among CHUU and CPHIV, respectively. Additionally, CPHIV with moderate versus low levels of SFA stearic acid (0.58 [0.03, 1.13]) exhibited a strong increase in EFI z‐score, whereas CPHIV and CHEU exhibited trending negative associations.

**FIGURE 2:**
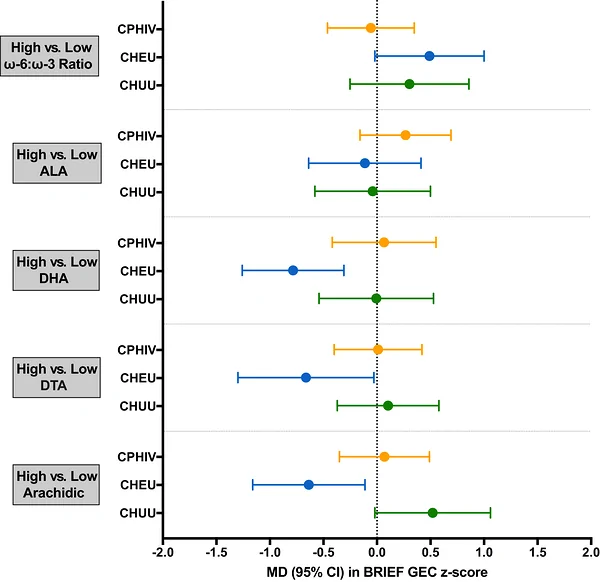
Time‐averaged HIV‐specific mean differences in proxy‐report Global Executive Composite z‐score by fatty acid tertiles. ALA, α‐linolenic acid; BRIEF, Behavior Rating Inventory of Executive Function; CHEU, children perinatally exposed to but uninfected with HIV; CHUU, children perinatally unexposed to and uninfected with HIV; CI, confidence interval; CPHIV, children with perinatal HIV infection; DHA, docosahexaenoic acid; DTA, docosatetraenoic acid; GEC, Global Executive Composite; MD, mean difference.

Figure 2 displays the exploratory GEC z‐score–FA tertile associations by perinatal HIV status. Among CHEU (MD [95% CI]: 0.49 [−0.02, 1.00]) and CHUU (0.30 [−0.25, 0.86]), those with a high versus low ω‐6:ω‐3 ratio exhibited a trending increase in GEC z‐score, whereas CPHIV exhibited a null association. High versus low ALA trended toward association with an increase in GEC z‐score among CPHIV and a decrease among CHEU and CHUU. CHEU with high versus low DHA exhibited a strong decrease in GEC z‐score (−0.78 [−1.26, −0.31]), whereas CPHIV and CHUU exhibited null associations. High DTA levels relative to low levels predicted strong decreases in GEC z‐score among CHEU (−0.66 [−1.30, −0.03]) and demonstrated null associations among CPHIV and CHUU. Additionally, CHEU with high versus low SFA arachidic acid levels demonstrated a decreased GEC z‐score (−0.64 [−1.16, −0.11]), whereas CHUU exhibited a trending positive association and CPHIV exhibited a null association.

### 3.6 Sensitivity Analyses

Sensitivity analyses using continuous FA measures yielded generally similar patterns of association compared with the main models. Several associations were attenuated when modeled linearly, suggesting potential nonmonotonic relationships between FAs and executive function (Table S2). Additional adjustment for HAZ did not largely alter MDs nor overall study conclusions (Table S3). Patterns of exploratory effect modification by perinatal HIV status were largely unchanged following additional HAZ adjustment but varied somewhat when FA measures were modeled continuously.

## 4 Discussion

In this study of 6–10‐year‐old Ugandan children, better executive function over time was associated with higher levels of total HUFAs, particularly ω‐6 HUFA arachidonic acid. Associations between FA levels and executive function further varied by perinatal HIV status. Among CHEU, executive function was strongly associated with PUFA, HUFA, and EFAD markers, whereas CPHIV and CHUU exhibited weaker or opposite associations.

The observation that higher total HUFA levels, particularly ω‐6 HUFA arachidonic acid, were associated with better executive function among all participants supports HUFAs’ supportive roles in neurocognitive development. The essential FAs ω‐6 LA and ω‐3 ALA are desaturated and elongated into their HUFA byproducts, which are more biologically active. These HUFAs serve as precursors to prostaglandins and eicosanoids, which are potent signaling molecules in the brain that enhance neurogenesis, synaptic function, and neuroinflammatory regulation [22]. Arachidonic acid, which constitutes 10%–12% of total FAs in the brain [14], increases neuronal membrane fluidity, synaptic plasticity, and neurotransmission [11, 13] and plays a central role in neuroimmune signaling [15, 16].

The physiological importance of arachidonic acid for child cognition has been demonstrated in randomized controlled trials and observational studies. In a randomized controlled trial of American children aged 4 months–1 year, arachidonic acid supplementation provided an extra cognitive benefit compared to DHA alone [18]. Observational studies have also linked higher arachidonic acid levels to improved executive function and behavioral outcomes, including better performance on executive function tasks among 2–6‐year‐old Ghanian children [17], lower prevalence of learning disorders among global child populations [20], and reduced conduct problems among 7–14‐year‐old Italian children [19]. Hence, the results presented here bolster previous evidence of positive associations between HUFAs, notably arachidonic acid, and cognition, specifically executive function, by demonstrating these associations persist over 12 months and apply to school‐aged children affected by perinatal HIV.

Associations between serum FAs and executive dysfunction varied by perinatal HIV status, suggesting meaningful heterogeneity in FA metabolism and neurodevelopmental vulnerability. Among CHUU, PUFA levels were not significantly associated with executive function. Rather, higher levels of long‐chain SFA arachidic acid trended toward association with greater executive dysfunction, whereas stearic acid showed an opposite, weaker relationship. These findings are partially consistent with experimental work linking SFA levels to reduced membrane integrity and increased neuroinflammation via Toll‐like receptor 4 activation [1, 3] and bolster epidemiologic evidence among 7–10‐year‐old American children that showed SFA intake was associated with impaired working memory/task switching [2]. These results suggest that SFA levels, rather than PUFA levels, may be more relevant for executive function among CHUU in this setting.

In contrast, CHEU demonstrated associations more consistent with hypothesized benefits of long‐chain PUFAs. Higher ω‐6 and ω‐3 HUFA levels were strongly associated with better executive function, aligning with prior studies linking increased DHA and DPA ω‐3 intake through fish to improved school performance, verbal learning, and reading comprehension among Danish and South African children [7, 9]. Importantly, this study generalizes these previous findings in the context of perinatal HIV exposure and infection. Additionally, a higher ω‐6:ω‐3 ratio was associated with worse executive function among CHEU, corroborating evidence that a higher plasma ω‐6:ω‐3 ratio was associated with worse working memory and planning task performance in 7–12‐year‐old American children [23]. Together, these findings suggest that CHEU may be particularly sensitive to an excess of ω‐6 PUFAs relative to ω‐3 PUFAs, which can lead to increased neuroinflammation [15].

Notably, among CHEU, higher levels of LA and total ω‐6 PUFA were associated with worse executive function, whereas higher levels of downstream ω‐6 HUFAs DTA and eicosadienoic acid were associated with better executive function. These associations suggest that executive function among CHEU may particularly benefit from the efficient conversion of LA into its downstream ω‐6 HUFAs. This interpretation is further supported by the observed associations between higher EFAD markers and better executive function among CHEU. EFAD markers measure decreased LA metabolism relative to increased ω‐9 metabolism to Mead acid [26] and may therefore indicate altered LA metabolism and downstream HUFA availability. Collectively, these results indicate that executive function among CHEU exhibits more beneficial associations with EFA metabolite levels than EFA levels themselves.

Among CPHIV, higher EFAD markers were associated, significantly and insignificantly, with worse executive functioning, consistent with study hypotheses and previous findings in Tanzanian children in which a lower T:T ratio was associated with better executive function [8]. The current study builds on this previous work by highlighting potential differences in associations by perinatal HIV status. Opposing associations among CPHIV and CHEU may reflect HIV‐related impairments in FA absorption and Δ‐6 desaturase and Δ‐5 desaturase activity, which would limit HUFA synthesis in CPHIV [42, 43], whereas CHEU may demonstrate preserved fat absorption and desaturase function [45]. Significant negative associations between long‐chain SFAs and executive dysfunction further distinguished CHEU from CPHIV, suggesting group‐specific differences in inflammatory processes or FA utilization [61]. Collectively, these findings warrant further study of nutritional interventions specific for perinatal HIV status and further investigation into how perinatal HIV exposure shapes FA metabolism and cognitive development.

There are limitations present in this study. First, these results lack generalizability outside of 6–10‐year‐old Ugandan children living in Kampala who are affected by perinatal HIV. Also, dietary intake data were not collected for study participants, and serum FA levels were only measured at baseline, providing limited ability to account for serum FA differences over 12 months. If children were fed consistently for 12 months, however, their serum FA levels should not significantly change. Executive function was only assessed using proxy‐reported questionnaire‐based measures, which introduces measurement and recall biases. Although the statistical analyses were adjusted for multiple caregiver variables and thus controlled for confounding, a more robust measure of executive function would also include performance‐based measures. Residual confounding is also present in this observational study, although statistical analyses were adjusted for potential confounders identified a priori based on the literature. Specifically, potential confounders such as caregiver‐report bias, inflammation, and diet could not be accounted for due to lack of data. Importantly, the influence of perinatal antiretroviral therapy exposure on associations between FAs and executive function was not examined in primary models because it is highly collinear with perinatal HIV status in this cohort, and the antiretroviral therapy exposure strata were insufficient to support stable estimation. However, future analyses with larger sample sizes should examine how perinatal antiretroviral therapy exposure among the different perinatal HIV exposure/infection groups may modify associations between serum FAs and executive function. Lastly, given the number of exposures, outcomes, and subgroup analyses, findings should be interpreted as hypothesis‐generating and may be subject to type I error due to multiple comparisons.

This study also contains several strengths. Perinatal HIV status was validated for study participants by performing PCR tests upon enrollment. All analyses, that is, population‐wide and pairwise comparisons, were conducted using a large sample size of over 200, which increases the validity of the presented results. Additionally, the executive function questionnaires were validated for use in this study population and were administered at three different time points, that is, baseline, 6‐, and 12‐months‐follow‐up, which increased the number of outcome measurements. The clustering of outcomes by individual participant and household, which can result in inaccurate data replication, was also controlled for in the statistical modeling presented. Serum FA levels were measured to assess FA intake, which may be a more objective measure of dietary FA intake versus traditional dietary assessment measures. The serum samples provided were also fasted, which increases their reliability as a marker of longer‐term FA intake [62]. Finally, sensitivity analyses using alternative exposure specifications and additional adjustment for child growth did not materially alter conclusions and support the robustness of the present findings. The categorical exposure specification in main models also allowed for the evaluation of potential nonmonotonic relationships that were not fully captured using a continuous exposure.

## 5 Conclusion

In conclusion, findings from 6‐ to 10‐year‐old children from Kampala, Uganda with varying levels of perinatal HIV exposure/infection support the beneficial associations of higher total HUFA and arachidonic acid levels with executive function. Executive function among CHEU demonstrated positive associations with HUFA levels, whereas CPHIV demonstrated negative associations with Mead acid levels. Future studies should explore how perinatal antiretroviral therapy and metabolic differences may complicate FA–executive function associations and use these associations to test targeted FA supplementation strategies among populations affected by perinatal HIV.

## Author Contributions

Vanessa N. Cardino, Bruno Giordani, Sarah K. Zalwango, and Amara E. Ezeamama performed the research. Vanessa N. Cardino, Jenifer I. Fenton, and Amara E. Ezeamama designed the research study. Sarah K. Zalwango, Jenifer I. Fenton, and Amara E. Ezeamama contributed essential reagents or tools. Vanessa N. Cardino, Alla Sikorskii, and Amara E. Ezeamama analyzed the data. Vanessa N. Cardino, Jenifer I. Fenton, and Amara E. Ezeamama wrote the paper. All the authors read and approved the final manuscript.

## Conflicts of Interest

The authors declare no conflicts of interest.

## Supporting information




**Supporting Information**: nyas70374‐sup‐0001‐TableS1‐S3.docx
