# Supplementary material for: Baseline Fatty Acids and Time‐Averaged Executive Function in Ugandan Children With Perinatal HIV
Source: Ann N Y Acad Sci. 2026 Aug 21;1562(1):e70374. doi: 10.1111/nyas.70374 (PMC13496357; doi:10.1111/nyas.70374)
Supplement: Supplementary file 1 [file NYAS-1562-0-s001.docx]

**Table S1.** Tertiles of baseline serum fatty acids by perinatal HIV status among 6-10-year-old Ugandan children

| **Fatty acid** |  | **Total**  **(n=243)** | **CPHIV**  **(n=84)** | | **CHEU**  **(n=78)** | | **CHUU**  **(n=81)** | |  |
| --- | --- | --- | --- | --- | --- | --- | --- | --- | --- |
|  | **Tertile** | **Range^†^** | **n** | **%** | **n** | **%** | **n** | **%** | **p-value** |
| EFAD markers |  |  |  |  |  |  |  |  |  |
| T:T ratio | Low | 0.00-0.00 | 20 | 23.81 | 30 | 38.46 | 31 | 38.27 | 0.08 |
|  | Moderate | 0.00-0.01 | 28 | 33.33 | 23 | 29.49 | 20 | 37.04 |  |
|  | High | 0.01-0.04 | 36 | 42.86 | 25 | 32.05 | 20 | 24.69 |  |
| Mead | Low | 0.00-0.05 | 18 | 21.43 | 32 | 41.03 | 31 | 38.27 | **<0.01** |
|  | Moderate | 0.06-0.09 | 25 | 29.76 | 26 | 33.33 | 30 | 37.04 |  |
|  | High | 0.09-0.53 | 41 | 48.81 | 20 | 25.64 | 20 | 24.69 |  |
| Arachidonic | Low | 4.18-10.81 | 19 | 22.62 | 32 | 41.03 | 30 | 37.04 | 0.06 |
|  | Moderate | 10.85-12.63 | 28 | 33.33 | 26 | 33.33 | 27 | 33.33 |  |
|  | High | 12.64-22.01 | 37 | 44.05 | 20 | 25.64 | 24 | 29.63 |  |
| Sums/ratios |  |  |  |  |  |  |  |  |  |
| HUFA ratio | Low | 4.40-14.65 | 30 | 35.71 | 24 | 30.77 | 27 | 33.33 | 0.70 |
|  | Moderate | 14.72-18.36 | 31 | 36.90 | 25 | 32.05 | 25 | 30.86 |  |
|  | High | 18.38-33.90 | 23 | 27.38 | 29 | 37.18 | 29 | 35.80 |  |
| Total HUFA | Low | 4.88-14.00 | 16 | 19.05 | 34 | 43.59 | 31 | 38.27 | **0.01** |
|  | Moderate | 14.03-16.47 | 32 | 38.10 | 22 | 28.21 | 27 | 33.33 |  |
|  | High | 16.49-33.93 | 36 | 42.86 | 22 | 28.21 | 23 | 28.40 |  |
| Omega 3 Index | Low | 0.60-2.70 | 23 | 27.38 | 26 | 33.33 | 32 | 39.51 | 0.56 |
|  | Moderate | 2.70-3.64 | 31 | 36.90 | 27 | 34.62 | 23 | 28.40 |  |
|  | High | 3.64-14.33 | 30 | 35.71 | 25 | 32.05 | 26 | 32.10 |  |
| Total ω-3 PUFA | Low | 0.50-2.38 | 24 | 28.57 | 28 | 35.90 | 29 | 35.80 | 0.83 |
|  | Moderate | 2.39-3.18 | 31 | 36.90 | 24 | 30.77 | 26 | 32.10 |  |
|  | High | 3.20-11.60 | 29 | 34.52 | 26 | 33.33 | 26 | 32.10 |  |
| Total ω-6 PUFA | Low | 28.36-47.43 | 31 | 36.90 | 24 | 30.77 | 26 | 32.10 | 0.89 |
|  | Moderate | 47.54-52.08 | 25 | 29.76 | 27 | 34.62 | 29 | 35.80 |  |
|  | High | 52.10-61.33 | 28 | 33.33 | 27 | 34.62 | 26 | 32.10 |  |
| ω-6:ω-3 ratio | Low | 4.58-15.14 | 31 | 36.90 | 25 | 32.05 | 25 | 30.86 | 0.94 |
|  | Moderate | 15.16-21.29 | 26 | 30.95 | 27 | 34.62 | 28 | 34.57 |  |
|  | High | 21.31-109.44 | 27 | 32.14 | 26 | 33.33 | 28 | 34.57 |  |
| Total PUFA | Low | 31.20-50.47 | 33 | 39.29 | 22 | 28.21 | 26 | 32.10 | 0.55 |
|  | Moderate | 50.51-54.96 | 24 | 28.57 | 27 | 34.62 | 30 | 37.04 |  |
|  | High | 55.00-64.76 | 27 | 32.14 | 29 | 37.18 | 25 | 30.86 |  |
| Total MUFA | Low | 2.22-5.10 | 28 | 33.33 | 26 | 33.33 | 27 | 33.33 | 0.98 |
|  | Moderate | 5.10-6.04 | 29 | 34.52 | 24 | 30.77 | 28 | 34.57 |  |
|  | High | 6.05-10.36 | 27 | 32.14 | 28 | 35.90 | 26 | 32.10 |  |
| Total SFA | Low | 31.33-39.45 | 25 | 29.76 | 29 | 37.18 | 27 | 33.33 | 0.65 |
|  | Moderate | 39.53-43.59 | 27 | 32.14 | 28 | 35.90 | 26 | 32.10 |  |
|  | High | 43.65-58.73 | 32 | 38.10 | 21 | 26.92 | 28 | 34.57 |  |
| ω-3 PUFA |  |  |  |  |  |  |  |  |  |
| ALA | Low | 0.01-0.17 | 30 | 35.71 | 26 | 33.33 | 25 | 30.86 | 0.96 |
|  | Moderate | 0.18-0.29 | 27 | 32.14 | 25 | 32.05 | 29 | 35.80 |  |
|  | High | 0.29-0.87 | 27 | 32.14 | 27 | 34.62 | 27 | 33.33 |  |
| EPA | Low | 0.07-0.32 | 29 | 34.52 | 28 | 35.90 | 24 | 29.63 | 0.62 |
|  | Moderate | 0.32-0.48 | 29 | 34.52 | 21 | 26.92 | 31 | 38.27 |  |
|  | High | 0.48-3.63 | 26 | 30.95 | 29 | 37.18 | 26 | 32.10 |  |
| DPA ω-3 | Low | 0.00-0.04 | 23 | 27.38 | 28 | 35.90 | 30 | 37.04 | 0.38 |
|  | Moderate | 0.04-0.07 | 27 | 32.14 | 29 | 37.18 | 25 | 30.86 |  |
|  | High | 0.07-0.20 | 34 | 40.48 | 21 | 26.92 | 26 | 32.10 |  |
| DHA | Low | 0.34-1.69 | 21 | 25.00 | 26 | 33.33 | 34 | 41.98 | 0.20 |
|  | Moderate | 1.69-2.35 | 33 | 39.29 | 27 | 34.62 | 21 | 25.93 |  |
|  | High | 2.36-7.53 | 30 | 35.71 | 25 | 32.05 | 26 | 32.10 |  |
| ω-6 PUFA |  |  |  |  |  |  |  |  |  |
| Linoleic | Low | 19.63-34.40 | 37 | 44.05 | 20 | 25.64 | 24 | 29.63 | 0.13 |
|  | Moderate | 34.53-39.58 | 25 | 29.76 | 28 | 35.90 | 28 | 34.57 |  |
|  | High | 39.58-51.04 | 22 | 26.19 | 30 | 38.46 | 29 | 35.80 |  |
| GLA | Low | 0.00-0.03 | 27 | 32.14 | 25 | 32.05 | 29 | 35.80 | **0.03** |
|  | Moderate | 0.03-0.07 | 19 | 22.62 | 33 | 42.31 | 29 | 35.80 |  |
|  | High | 0.07-0.28 | 38 | 45.24 | 20 | 25.64 | 23 | 28.40 |  |
| Eicosadienoic | Low | 0.05-0.17 | 21 | 25.00 | 29 | 37.18 | 31 | 38.27 | 0.18 |
|  | Moderate | 0.17-0.23 | 27 | 32.14 | 27 | 34.62 | 27 | 33.33 |  |
|  | High | 0.23-0.60 | 36 | 42.86 | 22 | 28.21 | 23 | 28.40 |  |
| DGLA | Low | 0.07-0.43 | 12 | 14.29 | 39 | 50.00 | 30 | 37.04 | **<0.01** |
|  | Moderate | 0.43-0.63 | 21 | 25.00 | 23 | 29.49 | 37 | 45.68 |  |
|  | High | 0.64-1.44 | 51 | 60.71 | 16 | 20.51 | 14 | 17.28 |  |
| DTA | Low | 0.00-0.01 | 22 | 26.19 | 31 | 39.74 | 28 | 34.57 | **0.02** |
|  | Moderate | 0.01-0.02 | 23 | 27.38 | 30 | 38.46 | 28 | 34.57 |  |
|  | High | 0.02-0.05 | 39 | 46.43 | 17 | 21.79 | 25 | 30.86 |  |
| DPA ω-6 | Low | 0.00-0.01 | 19 | 22.62 | 34 | 43.59 | 28 | 34.57 | **<0.01** |
|  | Moderate | 0.01-0.02 | 21 | 25.00 | 29 | 37.18 | 31 | 38.27 |  |
|  | High | 0.02-0.05 | 44 | 52.38 | 15 | 19.23 | 22 | 27.16 |  |
| MUFA |  |  |  |  |  |  |  |  |  |
| Palmitoleic | Low | 0.00-0.77 | 29 | 34.52 | 28 | 35.90 | 24 | 29.63 | 0.36 |
|  | Moderate | 0.77-1.13 | 33 | 39.29 | 21 | 26.92 | 27 | 33.33 |  |
|  | High | 1.13-3.34 | 22 | 26.19 | 29 | 37.18 | 30 | 37.04 |  |
| Palmitelaidic | Low | 0.01-0.05 | 27 | 32.14 | 25 | 32.05 | 29 | 35.80 | 0.13 |
|  | Moderate | 0.05-0.08 | 21 | 25.00 | 28 | 35.90 | 32 | 39.51 |  |
|  | High | 0.08-0.24 | 36 | 42.86 | 25 | 32.05 | 20 | 24.69 |  |
| Oleic | Low | 0.70-3.10 | 23 | 27.38 | 28 | 35.90 | 30 | 37.04 | 0.17 |
|  | Moderate | 3.11-3.63 | 25 | 29.76 | 25 | 32.05 | 31 | 38.27 |  |
|  | High | 3.63-6.20 | 36 | 42.86 | 25 | 32.05 | 20 | 24.69 |  |
| Eicosenoic | Low | 0.11-0.30 | 26 | 30.95 | 23 | 29.49 | 32 | 39.51 | 0.17 |
|  | Moderate | 0.30-0.36 | 23 | 27.38 | 32 | 41.03 | 26 | 32.10 |  |
|  | High | 0.36-4.67 | 35 | 41.67 | 23 | 29.49 | 23 | 28.40 |  |
| Nervonic | Low | 0.20-0.68 | 37 | 44.05 | 23 | 29.49 | 21 | 25.93 | 0.13 |
|  | Moderate | 0.68-0.86 | 22 | 26.19 | 28 | 35.90 | 31 | 38.27 |  |
|  | High | 0.86-1.88 | 25 | 29.76 | 27 | 34.62 | 29 | 35.80 |  |
| SFA |  |  |  |  |  |  |  |  |  |
| Myristic | Low | 0.05-0.49 | 28 | 33.33 | 30 | 38.46 | 23 | 28.40 | 0.29 |
|  | Moderate | 0.50-0.73 | 32 | 38.10 | 19 | 24.36 | 30 | 37.04 |  |
|  | High | 0.74-2.74 | 24 | 28.57 | 29 | 37.18 | 28 | 34.57 |  |
| Palmitic | Low | 19.85-27.60 | 32 | 38.10 | 28 | 35.90 | 21 | 25.93 | 0.28 |
|  | Moderate | 27.62-30.35 | 29 | 34.52 | 21 | 26.92 | 31 | 38.27 |  |
|  | High | 30.36-45.28 | 23 | 27.38 | 29 | 37.18 | 29 | 35.80 |  |
| Stearic | Low | 5.54-9.80 | 13 | 15.48 | 36 | 46.15 | 32 | 39.51 | **<0.01** |
|  | Moderate | 9.84-11.48 | 33 | 39.29 | 24 | 30.77 | 24 | 29.63 |  |
|  | High | 11.49-20.57 | 38 | 45.24 | 18 | 23.08 | 25 | 30.86 |  |
| Arachidic | Low | 0.04-0.16 | 18 | 21.43 | 30 | 38.46 | 33 | 40.74 | **0.04** |
|  | Moderate | 0.16-0.22 | 29 | 34.52 | 27 | 34.62 | 25 | 30.86 |  |
|  | High | 0.22-0.43 | 37 | 44.05 | 21 | 26.92 | 23 | 28.40 |  |
| Behenic | Low | 0.10-0.49 | 25 | 29.76 | 26 | 33.33 | 30 | 37.04 | 0.16 |
|  | Moderate | 0.49-0.64 | 26 | 30.95 | 33 | 42.31 | 22 | 27.16 |  |
|  | High | 0.65-2.04 | 33 | 39.29 | 19 | 24.36 | 29 | 35.80 |  |
| Lignoceric | Low | 0.02-0.25 | 24 | 28.57 | 31 | 39.74 | 26 | 32.10 | 0.54 |
|  | Moderate | 0.25-0.37 | 32 | 38.10 | 24 | 30.77 | 25 | 30.86 |  |
|  | High | 0.37-0.70 | 28 | 33.33 | 23 | 29.49 | 30 | 37.04 |  |

Abbreviations: ALA, α-linolenic acid; CHEU, children perinatally exposed to but uninfected with HIV; CHUU, children perinatally unexposed to and uninfected with HIV; CPHIV, children perinatally infected with HIV; DGLA, dihomo-γ-linolenic acid; DPA, docosapentaenoic acid; DTA, docosatetraenoic acid; EFAD, essential fatty acid deficiency; GLA, γ-linolenic acid; HUFA, highly unsaturated fatty acid; MUFA, monounsaturated fatty acid; PUFA, polyunsaturated fatty acid; SFA, saturated fatty acid; T:T ratio, triene:tetraene ratio

**^†^**All fatty acid ranges are reported as % of total fatty acids

**Table S2.** Sensitivity analyses using continuous serum fatty acid measures to evaluate associations with global executive function z-scores among 6-10-year-old Ugandan children

| **Fatty acid** | **BASC proxy-report Executive Functioning Index**  **z-score**  **(n=232)** | | | **BRIEF proxy-report Global Executive Composite z-score**  **(n=232)** | |  |
| --- | --- | --- | --- | --- | --- | --- |
|  | **Crude model** | **Adjusted model^†^** |  | **Crude model** | **Adjusted model^†^** |  |
|  | **β (95% CI)** | **β (95% CI)** | **FA*HIV**  **p-value** | **β (95% CI)** | **β (95% CI)** | **FA*HIV**  **p-value** |
| EFAD markers |  |  |  |  |  |  |
| T:T ratio | -0.06 (-0.17, 0.05) | -0.02 (-0.20, 0.16) | *0.03* | -0.05 (-0.13, 0.04) | -0.03 (-0.17, 0.10) | 0.74 |
| Mead | -0.10 (-0.22, 0.01) | -0.07 (-0.27, 0.13) | *0.02* | -0.09 (-0.18, -0.01) | -0.09 (-0.24, 0.06) | 0.88 |
| Arachidonic | -0.09 (-0.18, 0.00) | -0.08 (-0.21, 0.04) | 0.53 | -0.12 (-0.19, -0.05) | **-0.11 (-0.22, -0.01)** | 0.65 |
| Sums/ratios |  |  |  |  |  |  |
| HUFA ratio | 0.05 (-0.04, 0.14) | 0.05 (-0.09, 0.19) | 0.13 | -0.04 (-0.12, 0.05) | -0.01 (-0.14, 0.12) | 0.61 |
| Total HUFA | -0.07 (-0.15, 0.01) | -0.06 (-0.18, 0.06) | 0.89 | -0.12 (-0.19, -0.05) | -0.11 (-0.21, 0.00) | 0.63 |
| Omega-3 Index | 0.01 (-0.07, 0.10) | 0.01 (-0.11, 0.14) | 0.40 | -0.08 (-0.15, -0.01) | -0.05 (-0.17, 0.06) | 0.47 |
| Total ω-3 PUFA | 0.01 (-0.08, 0.09) | 0.01 (-0.12, 0.13) | 0.40 | -0.09 (-0.16, -0.02) | -0.06 (-0.18, 0.05) | 0.45 |
| Total ω-6 PUFA | 0.06 (-0.04, 0.16) | 0.04 (-0.11, 0.19) | *0.03* | 0.02 (-0.06, 0.09) | 0.00 (-0.11, 0.11) | 0.49 |
| ω-6:ω-3 ratio | 0.02 (-0.05, 0.10) | 0.01 (-0.11, 0.13) | 0.86 | 0.07 (-0.01, 0.16) | 0.04 (-0.09, 0.17) | 0.58 |
| Total PUFA | 0.06 (-0.03, 0.16) | 0.04 (-0.10, 0.19) | *0.06* | -0.01 (-0.08, 0.07) | -0.01 (-0.12, 0.09) | 0.53 |
| Total MUFA | -0.09 (-0.19, 0.00) | -0.08 (-0.22, 0.06) | 0.24 | -0.01 (-0.09, 0.06) | -0.02 (-0.13, 0.10) | 0.37 |
| Total SFA | -0.04 (-0.14, 0.05) | -0.03 (-0.16, 0.11) | *0.08* | 0.01 (-0.07, 0.09) | 0.02 (-0.08, 0.12) | 0.69 |
| ω-3 PUFA |  |  |  |  |  |  |
| ALA | -0.05 (-0.15, 0.05) | -0.05 (-0.21, 0.12) | 0.62 | -0.11 (-0.18, -0.03) | -0.08 (-0.20, 0.03) | 0.43 |
| EPA | 0.05 (-0.04, 0.14) | 0.05 (-0.09, 0.19) | 0.38 | -0.01 (-0.07, 0.06) | 0.00 (-0.10, 0.11) | 0.50 |
| DPA ω-3 | 0.01 (-0.09, 0.11) | 0.03 (-0.12, 0.17) | 0.23 | -0.04 (-0.12, 0.05) | -0.03 (-0.17, 0.10) | 0.11 |
| DHA | 0.00 (-0.08, 0.08) | 0.00 (-0.13, 0.12) | 0.41 | -0.10 (-0.17, -0.02) | -0.07 (-0.19, 0.05) | 0.29 |
| ω-6 PUFA |  |  |  |  |  |  |
| LA | 0.10 (0.01, 0.20) | 0.08 (-0.07, 0.24) | *0.02* | 0.07 (-0.01, 0.15) | 0.06 (-0.06, 0.18) | 0.31 |
| GLA | -0.02 (-0.13, 0.08) | 0.01 (-0.15, 0.16) | 0.98 | -0.04 (-0.14, 0.06) | -0.03 (-0.18, 0.12) | 0.24 |
| Eicosadienoic | -0.10 (-0.20, -0.01) | -0.07 (-0.23, 0.08) | *0.04* | -0.11 (-0.19, -0.03) | -0.10 (-0.22, 0.03) | 0.64 |
| DGLA | -0.07 (-0.15, 0.02) | -0.03 (-0.18, 0.12) | 0.19 | -0.08 (-0.16, 0.00) | -0.08 (-0.21, 0.06) | 0.82 |
| DTA | -0.09 (-0.19, 0.01) | -0.06 (-0.21, 0.10) | 0.17 | -0.04 (-0.12, 0.05) | -0.03 (-0.17, 0.11) | 0.72 |
| DPA ω-6 | -0.07 (-0.16, 0.02) | -0.03 (-0.19, 0.13) | 0.24 | -0.05 (-0.13, 0.03) | -0.02 (-0.15, 0.11) | 0.17 |
| MUFA |  |  |  |  |  |  |
| Palmitoleic | -0.09 (-0.18, 0.00) | -0.07 (-0.21, 0.07) | 0.57 | 0.03 (-0.05, 0.11) | 0.03 (-0.09, 0.16) | 0.58 |
| Palmitelaidic | -0.09 (-0.20, 0.01) | 0.05 (-0.22, 0.12) | *0.05* | -0.05 (-0.13, 0.02) | -0.04 (-0.16, 0.09) | 0.62 |
| Oleic | -0.10 (-0.20, -0.01) | -0.09 (-0.22, 0.04) | 0.67 | -0.04 (-0.12, 0.03) | -0.04 (-0.15, 0.07) | 0.40 |
| Eicosenoic | 0.04 (0.00, 0.08) | 0.05 (-0.10, 0.19) | 0.25 | -0.03 (-0.06, 0.01) | -0.05 (-0.10, 0.00) | 0.60 |
| Nervonic | 0.02 (-0.08, 0.11) | 0.01 (-0.13, 0.14) | 0.72 | 0.04 (-0.03, 0.12) | 0.04 (-0.07, 0.16) | *0.06* |
| SFA |  |  |  |  |  |  |
| Myristic | -0.09 (-0.19, 0.00) | -0.05 (-0.19, 0.09) | 0.35 | -0.05 (-0.12, 0.03) | -0.02 (-0.13, 0.08) | 0.47 |
| Palmitic | -0.03 (-0.13, 0.06) | -0.03 (-0.17, 0.10) | 0.17 | 0.04 (-0.04, 0.11) | 0.04 (-0.07, 0.14) | 0.71 |
| Stearic | -0.03 (-0.12, 0.06) | 0.00 (-0.13, 0.12) | 0.14 | -0.04 (-0.12, 0.04) | -0.02 (-0.12, 0.08) | 0.68 |
| Arachidic | -0.06 (-0.16, 0.03) | -0.04 (-0.17, 0.09) | 0.94 | 0.03 (-0.05, 0.12) | 0.05 (-0.08, 0.18) | *0.02* |
| Behenic | 0.00 (-0.08, 0.08) | 0.00 (-0.11, 0.12) | 0.51 | 0.06 (-0.02, 0.14) | 0.06 (-0.06, 0.18) | 0.43 |
| Lignoceric | 0.00 (-0.08, 0.09) | 0.02 (-0.10, 0.14) | 0.43 | 0.03 (-0.05, 0.11) | 0.05 (-0.07, 0.17) | 0.28 |

Abbreviations: ALA, α-linolenic acid; BASC, Behavior Assessment System for Children; BRIEF, Behavior Rating Inventory for Executive Function; CI, confidence interval; DGLA, dihomo-γ-linolenic acid; DHA, docosahexaenoic acid; DPA, docosapentaenoic acid; DTA, docosatetraenoic acid; EFAD, essential fatty acid deficiency; EPA, eicosapentaenoic acid; GLA, γ-linolenic acid; HUFA, highly unsaturated fatty acid; LA, linoleic acid; MD, mean difference; MUFA, monounsaturated fatty acid; PUFA, polyunsaturated fatty acid; SFA, saturated fatty acid; T:T ratio, triene:tetraene ratio

Beta values represent the change in global executive function z-score for every standard deviation increase in fatty acid measure

**Table S3.** Sensitivity analyses of time-averaged mean differences in global executive function z-scores relative to low serum fatty acid tertile among 6-10-year-old Ugandan children with additional adjustment for height-for-age z-score

| **Fatty acid** |  | **BASC proxy-report Executive Functioning Index z-score**  **(n=232)** | | **BRIEF proxy-report Global Executive Composite z-score**  **(n=232)** | |
| --- | --- | --- | --- | --- | --- |
|  | **Tertile** | **Main model** | **HAZ-adjusted model** | **Main model** | **HAZ-adjusted model** |
|  |  | **MD (95% CI)** | **MD (95% CI)** | **MD (95% CI)** | **MD (95% CI)** |
| EFAD markers |  |  |  |  |  |
| T:T ratio | Moderate | - | - | 0.01 (-0.26, 0.27) | 0.00 (-0.26, 0.26) |
|  | High | - | - | -0.06 (-0.35, 0.23) | -0.09 (-0.38, 0.21) |
| Mead | Moderate | - | - | -0.02 (-0.29, 0.24) | -0.03 (-0.30, 0.23) |
|  | High | - | - | -0.05 (-0.36, 0.26) | -0.06 (-0.38, 0.25) |
| Arachidonic | Moderate | **-0.40 (-0.71, -0.09)** | **-0.37 (-0.69, -0.06)** | **-0.30 (-0.58, -0.03)** | -0.28 (-0.55, 0.00) |
|  | High | -0.23 (-0.55, 0.10) | -0.24 (-0.56, 0.08) | **-0.31 (-0.61, -0.01)** | **-0.31 (-0.61, -0.02)** |
| Sums/ratios |  |  |  |  |  |
| HUFA ratio | Moderate | -0.15 (-0.45, 0.15) | 0.17 (-0.46, 0.13) | -0.16 (-0.45, 0.13) | -0.16 (-0.45, 0.13) |
|  | High | -0.03 (-0.38, 0.32) | -0.05 (-0.39, 0.29) | -0.09 (-0.38, 0.20) | -0.12 (-0.41, 0.17) |
| Total HUFA | Moderate | **-0.41 (-0.72, -0.11)** | **-0.38 (-0.69, -0.08)** | -0.26 (-0.55, 0.03) | -0.23 (-0.52, 0.06) |
|  | High | -0.19 (-0.54, 0.15) | -0.20 (-0.54, 0.15) | -0.24 (-0.56, 0.08) | -0.24 (-0.56, 0.08) |
| Omega-3 Index | Moderate | -0.23 (-0.53, 0.06) | -0.22 (-0.52, 0.07) | -0.13 (-0.41, 0.14) | -0.12 (-0.39, 0.15) |
|  | High | -0.09 (-0.41, 0.23) | -0.10 (-0.41, 0.22) | -0.15 (-0.43, 0.13) | -0.17 (-0.45, 0.11) |
| Total ω-3 PUFA | Moderate | -0.25 (-0.54, 0.05) | -0.23 (-0.53, 0.06) | -0.20 (-0.47, 0.07) | -0.18 (-0.44, 0.09) |
|  | High | -0.08 (-0.39, 0.24) | -0.08 (-0.40, 0.23) | -0.21 (-0.50, 0.08) | -0.22 (-0.51, 0.06) |
| Total ω-6 PUFA | Moderate | - | - | -0.06 (-0.33, 0.22) | -0.02 (-0.29, 0.26) |
|  | High | - | - | 0.07 (-0.21, 0.35) | 0.11 (-0.17, 0.40) |
| ω-6:ω-3 ratio | Moderate | 0.03 (-0.32, 0.38) | 0.02 (-0.32, 0.36) | - | 0.11 (-0.18, 0.41) |
|  | High | 0.15 (-0.18, 0.48) | 0.16 (-0.17, 0.48) | - | 0.26 (-0.02, 0.54) |
| Total PUFA | Moderate | - | - | 0.06 (-0.22, 0.33) | 0.06 (-0.22, 0.33) |
|  | High | - | - | 0.01 (-0.25, 0.28) | 0.10 (-0.17, 0.37) |
| Total MUFA | Moderate | 0.00 (-0.30, 0.29) | -0.01 (-0.30, 0.28) | 0.06 (-0.20, 0.32) | 0.06 (-0.20, 0.32) |
|  | High | -0.13 (-0.47, 0.20) | -0.15 (-0.48, 0.19) | 0.02 (-0.27, 0.32) | 0.01 (-0.28, 0.31) |
| Total SFA | Moderate | 0.07 (-0.25, 0.38) | 0.06 (-0.25, 0.37) | -0.02 (-0.29, 0.25) | -0.01 (-0.28, 0.27) |
|  | High | -0.03 (-0.35, 0.30) | -0.07 (-0.39, 0.26) | 0.05 (-0.22, 0.31) | 0.02 (-0.25, 0.29) |
| ω-3 PUFA |  |  |  |  |  |
| ALA | Moderate | -0.14 (-0.46, 0.17) | -0.16 (-0.48, 0.16) | - | - |
|  | High | 0.01 (-0.32, 0.33) | 0.01 (-0.32, 0.33) | - | - |
| EPA | Moderate | 0.00 (-0.34, 0.34) | -0.02 (-0.35, 0.32) | 0.00 (-0.31, 0.30) | -0.01 (-0.32, 0.30) |
|  | High | 0.10 (-0.25, 0.44) | 0.09 (-0.25, 0.43) | -0.04 (-0.34, 0.25) | -0.05 (-0.34, 0.24) |
| DPA ω-3 | Moderate | 0.10 (-0.20, 0.40) | 0.07 (-0.23, 0.37) | 0.08 (-0.18, 0.35) | 0.07 (-0.20, 0.33) |
|  | High | 0.04 (-0.30, 0.38) | 0.01 (-0.33, 0.34) | -0.06 (-0.34, 0.23) | -0.09 (-0.38, 0.19) |
| DHA | Moderate | -0.20 (-0.49, 0.10) | -0.18 (-0.48, 0.12) | - | - |
|  | High | -0.17 (-0.50, 0.15) | -0.19 (-0.51, 0.14) | - | - |
| ω-6 PUFA |  |  |  |  |  |
| LA | Moderate | - | - | -0.03 (-0.32, 0.26) | 0.01 (-0.28, 0.31) |
|  | High | - | - | 0.13 (-0.18, 0.43) | 0.16 (-0.13, 0.46) |
| GLA | Moderate | -0.04 (-0.36, 0.28) | 0.00 (-0.32, 0.31) | 0.03 (-0.27, 0.33) | 0.06 (-0.23, 0.35) |
|  | High | 0.05 (-0.25, 0.35) | 0.08 (-0.22, 0.39) | -0.06 (-0.35, 0.23) | -0.03 (-0.32, 0.26) |
| Eicosadienoic | Moderate | - | - | 0.03 (-0.22, 0.28) | 0.02 (-0.23, 0.28) |
|  | High | - | - | -0.16 (-0.44, 0.13) | -0.17 (-0.45, 0.12) |
| DGLA | Moderate | -0.13 (-0.44, 0.17) | -0.12 (-0.42, 0.18) | -0.05 (-0.31, 0.20) | -0.03 (-0.29, 0.23) |
|  | High | -0.12 (-0.52, 0.29) | -0.11 (-0.51, 0.29) | -0.17 (-0.51, 0.17) | -0.16 (-0.50, 0.18) |
| DTA | Moderate | - | - | - | - |
|  | High | - | - | - | - |
| DPA ω-6 | Moderate | -0.01 (-0.32, 0.31) | 0.00 (-0.32, 0.31) | -0.03 (-0.29, 0.22) | -0.03 (-0.29, 0.23) |
|  | High | -0.17 (-0.51, 0.18) | -0.18 (-0.53, 0.16) | -0.25 (-0.55, 0.05) | -0.27 (-0.57, 0.03) |
| MUFA |  |  |  |  |  |
| Palmitoleic | Moderate | -0.02 (-0.33, 0.30) | -0.03 (-0.35, 0.29) | 0.05 (-0.21, 0.31) | 0.05 (-0.21, 0.31) |
|  | High | -0.13 (-0.48, 0.21) | -0.13 (-0.48, 0.22) | 0.18 (-0.12, 0.48) | 0.19 (-0.11, 0.49) |
| Palmitelaidic | Moderate | 0.14 (-0.16, 0.45) | - | 0.04 (-0.24, 0.31) | 0.02 (-0.26, 0.29) |
|  | High | -0.05 (-0.40, 0.29) | - | -0.08 (-0.37, 0.21) | -0.09 (-0.38, 0.20) |
| Oleic | Moderate | -0.06 (-0.37, 0.25) | -0.05 (-0.35, 0.26) | 0.04 (-0.24, 0.31) | 0.05 (-0.22, 0.32) |
|  | High | -0.14 (-0.46, 0.18) | -0.17 (-0.49, 0.15) | -0.01 (-0.30, 0.28) | -0.04 (-0.32, 0.25) |
| Eicosenoic | Moderate | 0.10 (-0.20, 0.41) | 0.10 (-0.21, 0.40) | 0.11 (-0.18, 0.41) | 0.12 (-0.18, 0.42) |
|  | High | -0.07 (-0.39, 0.26) | -0.10 (-0.42, 0.23) | -0.04 (-0.33, 0.26) | -0.07 (-0.37, 0.22) |
| Nervonic | Moderate | -0.13 (-0.43, 0.17) | -0.16 (-0.45, 0.14) | 0.00 (-0.28, 0.27) | -0.04 (-0.32, 0.24) |
|  | High | 0.02 (-0.29, 0.33) | -0.02 (-0.32, 0.28) | 0.15 (-0.12, 0.43) | 0.12 (-0.16, 0.39) |
| SFA |  |  |  |  |  |
| Myristic | Moderate | 0.02 (-0.30, 0.35) | -0.01 (-0.34, 0.32) | -0.20 (-0.47, 0.07) | -0.23 (-0.49, 0.04) |
|  | High | -0.17 (-0.51, 0.17) | -0.15 (-0.49, 0.18) | -0.11 (-0.40, 0.17) | -0.09 (-0.38, 0.20) |
| Palmitic | Moderate | 0.00 (-0.31, 0.32) | 0.00 (-0.31, 0.31) | -0.02 (-0.29, 0.25) | -0.01 (-0.28, 0.26) |
|  | High | 0.04 (-0.30, 0.38) | 0.01 (-0.33, 0.34) | 0.10 (-0.17, 0.38) | 0.07 (-0.21, 0.35) |
| Stearic | Moderate | - | - | -0.15 (-0.43, 0.13) | -0.19 (-0.47, 0.09) |
|  | High | - | - | -0.07 (-0.35, 0.22) | -0.12 (-0.41, 0.17) |
| Arachidic | Moderate | 0.00 (-0.30, 0.30) | -0.06 (-0.36, 0.24) | - | - |
|  | High | -0.18 (-0.49, 0.13) | -0.25 (-0.56, 0.07) | - | - |
| Behenic | Moderate | -0.03 (-0.36, 0.30) | -0.05 (-0.38, 0.28) | 0.17 (-0.09, 0.44) | 0.16 (-0.11, 0.42) |
|  | High | -0.21 (-0.52, 0.11) | -0.24 (-0.55, 0.08) | 0.03 (-0.25, 0.30) | 0.01 (-0.28, 0.29) |
| Lignoceric | Moderate | -0.02 (-0.33, 0.29) | -0.06 (-0.37, 0.25) | 0.04 (-0.23, 0.31) | 0.00 (-0.26, 0.26) |
|  | High | 0.04 (-0.27, 0.36) | 0.01 (-0.31, 0.32) | 0.07 (-0.21, 0.35) | 0.03 (-0.25, 0.32) |

Abbreviations: ALA, α-linolenic acid; BASC, Behavior Assessment System for Children; BRIEF, Behavior Rating Inventory for Executive Function; CI, confidence interval; DGLA, dihomo-γ-linolenic acid; DHA, docosahexaenoic acid; DPA, docosapentaenoic acid; DTA, docosatetraenoic acid; EFAD, essential fatty acid deficiency; EPA, eicosapentaenoic acid; GLA, γ-linolenic acid; HAZ; height-for-age z-score; HUFA, highly unsaturated fatty acid; LA, linoleic acid; MD, mean difference; MUFA, monounsaturated fatty acid; PUFA, polyunsaturated fatty acid; SFA, saturated fatty acid; T:T ratio, triene:tetraene ratio

Missing values represent associations that had a significant perinatal HIV status*fatty acid (p<0.10)
